# SEX AND THE BIOPSYCHOSOCIAL MODEL: SEXUAL ACTIVITY AND GENDER DISPARITY IN OLDER ADULTS

**DOI:** 10.1093/geroni/igz038.1109

**Published:** 2019-11-08

**Authors:** Rebecca Reinhardt, Zaver D Moore, Alexandria Nuccio

**Affiliations:** Nova Southeastern University, Fort Lauderdale, Florida, United States

## Abstract

The biopsychosocial model emphasizes relational factors such as quality and availability as key components to older adult sexual activity (Gillespie, 2017). Supporting these findings, a previous study found that older adults aged 65 and over reported having more sex in the past six months but fewer sexual partners in the past year than younger adults. The current study seeks to further explore sexual activity by gender specifically, number of sex partners in the last year, and frequency of sex over the past six months in older adults. To better understand sexual activity and gender differences, 499 (male=59.7%, female 40.3%) participants aged 65 to 93 were selected from the de-identified Survey of Midlife in the US database (MIDUS-3). A Weltch T-test was used examining sexual activity among older adults based on gender. Results indicated that the number of sex partners within the past year was similar for females (M=1.10, SD=.49) and males (M=1.04, SD=.28), t(477)=1.62, p=.10, d^=.07. Further, results indicated sex frequency within the last six months was similar between females (M=3.41, SD=1.55) and males (M=3.66, SD=1.48), t(415)=1.76, p=.08, d^=.14. Contrary to previous research, the present findings suggest there are no gender differences in number of sex partners or sex frequency for older adults. The current findings draw attention to potential discrepancies within this under-explored subject area. While implications of these findings can improve communication regarding sexual health, future research should focus on how aspects of the biopsychosocial model can be a protective factor for the sexual health of older adults.

